# The relationship between HbA1c control pattern and atherosclerosis progression of diabetes: a prospective study of Chinese population

**DOI:** 10.1186/s13098-024-01370-4

**Published:** 2024-06-10

**Authors:** Kun Li, Longyan Yang, Dong Zhao

**Affiliations:** 1 Center for Endocrine Metabolism and Immune Diseases, Beijing Luhe Hospital, Capital Medical University, Beijing, 101149 China; 2 Beijing Key Laboratory of Diabetes Research and Care, No.82, Xinhua South Road, Beijing, 101149 China

**Keywords:** Atherosclerosis, Diabetes, HbA1c, Cohort

## Abstract

**Background:**

This study aims to comprehensively explain of glycosylated Hemoglobin (HbA1c) control patterns and help determine the causal relationship between glycemic control patterns and atherosclerosis progression, thereby contributing to the effective management of diabetes complications.

**Method:**

All participants registered at the National Metabolic Management Center (MMC) of Beijing Luhe Hospital. The HbA1c pattern was described by HbA1c variability and trajectory groups of HbA1c. Then we examined the associations between the HbA1c pattern and the changes of intima-media thickness (ΔIMT) using covariate-adjusted means (SE) of ΔIMT, which were calculated by multiple linear regression analyses adjusted for the covariates. Finally, a cross-lagged panel model (CLPM) was performed to further verify the bidirectional relationship between IMT and HbA1c.

**Results:**

After data cleaning, a total of 1041 type 2 diabetes patients aged 20–80 years were included in this study. Except for average real variability (ARV), the other variation variables of HbA1c were associated with ΔIMT% (P < 0.05). Four discrete trajectories of HbA1c were identified in trajectory analysis. Comparing with the low-stable trajectory group of HbA1c, the covariate-adjusted means (SE) of ΔIMT% were significantly higher in Moderate-increase, U-shape and relative high trajectory groups, the mean (SE) were 7.03 (0.031), 15.49 (0.185), 14.15 (0.029), respectively. Meanwhile, there were significant bidirectional cross-lagged associations between HbA1c and IMT after adjusting for covariates.

**Conclusion:**

We found four discrete trajectory groups of HbA1c during the long-term follow-up of diabetes. There was a positive association between HbA1c variability and the progression of atherosclerosis. Our study suggested that patients with diabetes should avoid roller coaster changes in glucose over a long period when controlling blood glucose.

**Supplementary Information:**

The online version contains supplementary material available at 10.1186/s13098-024-01370-4.

## Introduction

Type 2 diabetes mellitus (T2DM) is one of the most significant health problem [1]. In a nationally representative cross-sectional study conducted in mainland China with 173,642 participants in 2018, the estimated prevalence of diabetes was 12.4%, and of prediabetes was 38.1% [2]. Vascular complications of diabetes can lead to disability of diabetic patients and further affect the quality of life of patients [3–5]. A recent epidemiologic study has shown that cardiovascular disease (CVD) has climbed to the leading cause of mortality and morbidity in diabetes patients [6].

Carotid artery intima-media thickness (IMT), a noninvasive imaging marker of carotid atherosclerosis, has become an essential tool in evaluating and monitoring early atherosclerotic vascular changes and the progression of CVD [7]. In the last decade, several studies reported an association between glycemic level and cardiovascular outcomes [8, 9]. In addition, a retrospective study has found an association between short-term glycemic variability assessed using continuous glucose monitoring (CGM) and subclinical atherosclerosis in T2DM patients [10].

Despite these findings, there is currently a lack of significant evidence to describe the relationship between the HbA1c control pattern and the cardiovascular progression of diabetes. During the long-term treatment of diabetes patients, the glycemic status, as evidenced by the temporal fluctuations within an individual and inter-individual differences in blood glucose control. When interpreting blood glucose data, individual-level analysis encompasses single time point measurements, trends between two consecutive blood glucose measurements, and variability across multiple tests. At the group level, clustering patterns of blood glucose changes can be employed to describe collective modes of blood glucose control [5, 11–14].

Therefore, this study aimed to elucidate the causal relationship between HbA1c control patterns and the progression of atherosclerosis using long-term glucose monitoring data in patients with diabetes. All participants of this study have been registered at the National Metabolic Management Center (MMC), an innovation project for the management of metabolic diseases and complications in China [15]. We hope the present study will provide a comprehensive description of HbA1c control pattern and help determine the causal relationship between glycemic control patterns and atherosclerosis progression, thereby contributing to the effective management of diabetes complications.

## Method

### Study design and population

From October 2017 to April 2023, all participants of this prospective study were registered at the Metabolic Management Center (MMC) of Beijing Luhe Hospital. The MMC is a national project that aims to manage metabolic patients according to a standardized approach. All MMCs in China have the same facilities structure, databases, and layout, as well as the same routine daily operations, which aims to establish a platform with standardized diagnosis and treatment of metabolic diseases and their long-term follow-up. Patients can get one-stop care to receive a comprehensive series of services from registration, tests, evaluation, prescriptions, to health education. The protocol of the MMC project was published previously [15].

Patients with type 2 diabetes aged 18–80 years were recruited. The recruitment process included blood sample collection, systematic physical examination, and oral questionnaire interviews. Diabetes was defined as having a fasting plasma glucose level of 126.13 mg/dL, a 2-h plasma glucose level of at least 200 mg/dL, an HbA1c level of at least 6.5%, or a self-reported previous diagnosis by health care professionals. In this study, only type 2 diabetes (T2DM) were enrolled.

Participants were excluded from the study if they met any of the following criteria: (1) pregnant or nursing women; (2) suffering from a malignant tumor; (3) experiencing acute complications of diabetes; (4) had visited times less than four times and the follow-up time less than 12 months; (5) had missing data of critical variables.

The study protocol was approved by the Medical Ethics Committee of Beijing Luhe Hospital, Capital Medical University. This study was performed by the Declaration of Helsinki, and all participants provided written informed consent.

### Measurement of HbA1c and IMT

Blood samples were obtained in the morning after overnight fasting. Glycated hemoglobin (HbA1c) levels were assayed using the method of high-performance liquid chromatography (HPLC) with a D10 set (Bio-RAD, Hercules, CA, USA). HbA1c was a normal inspection in the MMC program, which was examined every 3–6 months for each participant. The average time for HbA1c measurements in this study was 5.07 ± 2.25 months.

Participants were examined by ultrasonography. Participants in the supine position with the head slightly extended and turned to the opposite direction of the carotid artery being studied. Images were recorded at the internal carotid arteries bilaterally. The maximum carotid IMT readings of the right and left far walls for common, bulb, and internal segments were used for analysis. Considering the study design and statistical methods of this study, only the IMT results of the first and last follow-up were included in the analysis.

### Covariates

Systematic physical examination and oral questionnaire interviews were performed by trained personnel according to the protocol. The questionnaire contains information on demographic characteristics (including age, sex, education level, weight, height), medical history (including duration of diabetes, hypertension history, antihypertensive drugs usage, lipids treatments information.), and lifestyle factors (including cigarette smoking, drinking.). Smoking status was defined as ‘ideal’ if the participants did not smoke or had quit smoking for more than 12 months. Drinking status was recorded as ‘yes’ for participants who drank weekly or almost weekly. Education attainment was categorized as less than high school and high school or more.

Height and body weight were measured with a standard protocol, and body mass index (BMI) was calculated as weight divided by height squared. Moreover, an auto biochemical analyzer measured LDL, HDL, and triglyceride (AU5800, Beckman Coulter, USA).

### Statistical analysis

Continuous variables were described as mean ± standard deviation (SD) or median [interquartile range (IQR)], and categorical variables were described as frequency (%). Logarithmically transformed were required before statistical analysis when data were tested as non-normal distribution. We used the Cochran-Armitage trend test and linear regression analysis to calculate the P values for categorical and continuous variables across the groups.

The coefficients of variation (CV) of HbA1c were calculated as the ration of standard deviation (SD) to the mean. The ARV (average real variability) was calculated as the average of the absolute differences between consecutive HbA1c measurements (Formulate 1) [16].

1 $$ARV = \frac{{\Sigma_{k = 1}^{N - 1} \left| {HbA1c_{K + 1} - HbA1c_{K} } \right|}}{N - 1}$$

where K is the ordinal number of HbA1c, and N is the total number of HbA1c measurements.

The variation independent of the mean (VIM) was calculated as the SD divided by the mean HbA1c raised to the power of x, where x is obtained from fitting a nonlinear regression model (Formulate 2) [17].

2 $$VIM = \frac{{k*Standard \,Deviation \,\left( {HbA1c} \right)}}{{Mean\left( {HbA1c} \right)^{x} }}\,where\,k = Mean\,\left( {Mean(SBP)} \right)^{x}$$

Multiple linear regressions were used to explore the association between the variation of HbA1c and the changes of IMT; two models were established and adjusted for different covariates. Model 1 was adjusted by age, duration of diabetes and sex; model 2 was adjusted by ideal smoking, drink status, BMI, education level, SBP, LDL, HDL, and TC plus the covariates in model 1; model 3 was adjusted by oral antidiabetic agents, lipid-lowering agents and insulin plus the covariates in model 2. As a secondary analysis, we performed generalized additive models (GAM) to investigate the association between variation of HbA1c and the changes of IMT in non-linear conditions.

The latent class mixture model (LCMM) was used to identify similar trajectories of HbA1c [3, 12–14]. The *lcmm* package of R was used to execute the procedure, with the number of latent categories ranging from 2 to 5. Each trajectory group was named based on the baseline of HbA1c and the visual change patterns observed of HbA1c during the follow-up period. The optimal model was selected using the Bayesian information criterion (BIC).

We calculated the change value of IMT (ΔIMT) and the change percent of IMT (ΔIMT%) of every participant in the whole follow-up period as the primary outcome. The associations between each HbA1c trajectory pattern and ΔIMT% were examined using covariate-adjusted means (SE) of ΔIMT and ΔIMT%, which were calculated by multiple linear regression analyses adjusted for the covariates mentioned previously.

Finally, a cross-lagged panel model (CLPM) was performed to further verify the bidirectional relationship between IMT and HbA1c, which measured the effect size of baseline HbA1c measurement on subsequent IMT and the effect size of baseline IMT on subsequent HbA1c measurement simultaneously [18–20]. The analysis procedure was performed in the *lavaan* package of R. The CLPM is adjusted by age, sex, duration of diabetes, SBP, ideal smoking, drink status, HDL, LDL, oral antidiabetic agents, lipid-lowering agents and insulin.

Moreover, we also performed sex subgroup analyses to investigate the consistency of the association between variation of HbA1c and the changes of IMT. Multiple linear regressions adjusted all covariables in model 2.

All statistical analyses were performed in R software (version 4.2.2, https://www.r-project.org/).

## Result

### Demographic characteristics

**Fig. 1:**
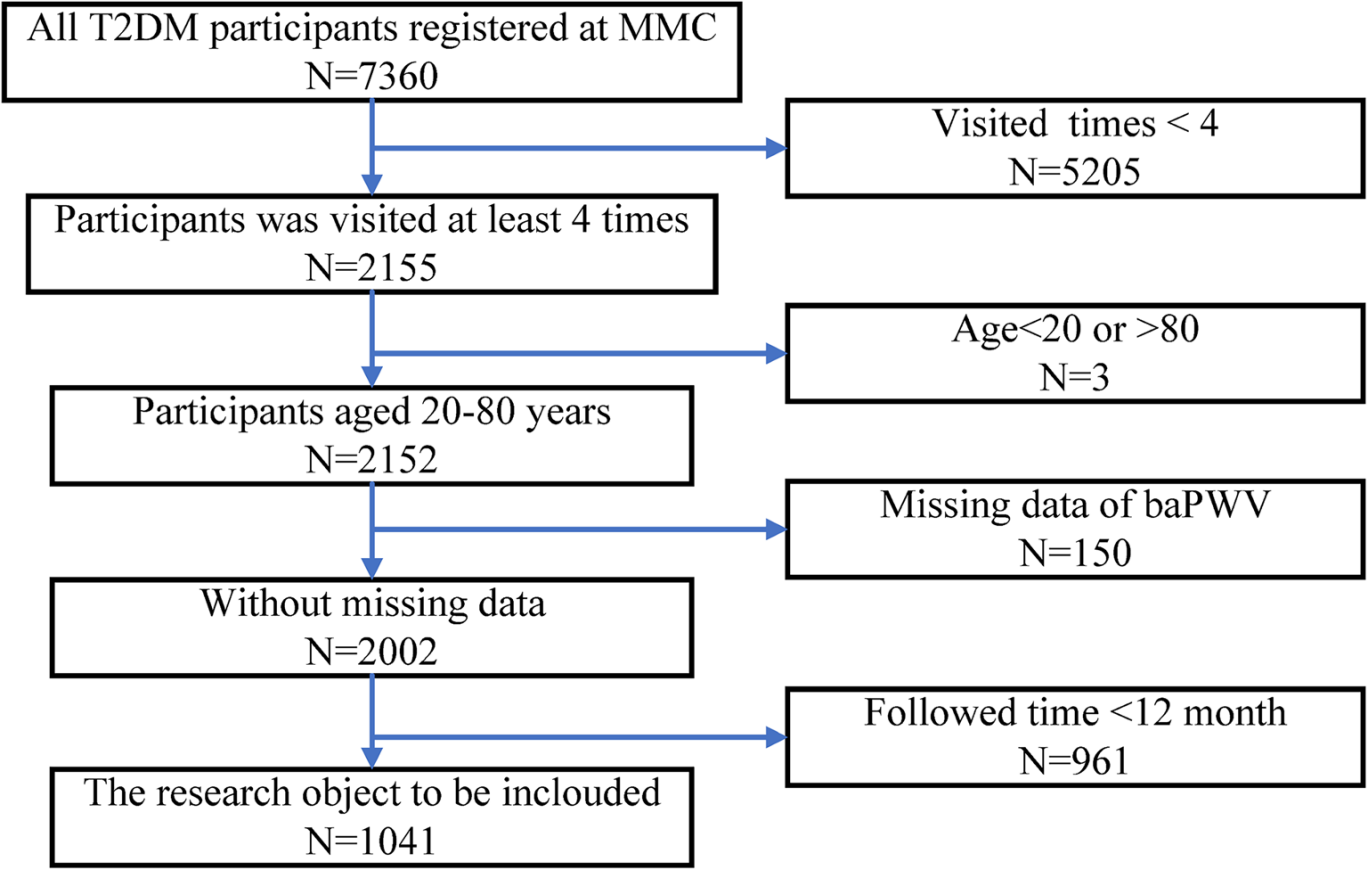
The data cleaning procedure of this study

There were 7360 T2DM patients registered at MMC from October 2017 to April 2023. The data cleaning procedure was performed to detect missing values and abnormal values. Participants were screened according to the inclusion and exclusion criteria. The whole data cleaning procedure could be seen in Fig. 1. After data cleaning, there were 1041 participants enrolled in this study. Participants’ mean (SD) age was 51.27 (11.81) years, and 605 (58.11%) were males. The mean follow-up time was 28.78 months. More demographic and clinical characteristics of participants were shown in Table 1.

**Table 1:** Demographic characteristics of participants for the first visit of this study

	ALL	male	female	p
	*N* = *1041*	*N* = *605*	*N* = *436*	
Duration,mean(SD),month	84.06 (84.96)^a^	77.12 (82.29)	93.80 (87.74)	0.003
Age,mean(SD),year	51.27 (11.81)	49.38 (11.60)	53.88 (11.60)	< 0.001
Ideal Smoking,n(%)	315 (30.41%)	294 (48.84%)	21 (4.84%)	< 0.001
Drink status,n(%)	435 (42.07%)	396 (65.89%)	39 (9.01%)	< 0.001
High school or more, n (%)	625 (60.04%)	401 (66.28%)	224 (51.38%)	< 0.001
Weight,mean(SD),kg	74.52 (14.19)	80.33 (13.49)	66.47 (10.83)	< 0.001
BMI,mean(SD),kg/m2	26.82 (4.00)	27.20 (3.99)	26.31 (3.95)	< 0.001
height,mean(SD),cm	166.34 (8.62)	171.72 (6.18)	158.89 (5.36)	< 0.001
SBP,mean(SD),mmHg	133.34 (18.00)	132.69 (17.68)	134.23 (18.42)	0.176
DBP,mean(SD),mmHg	80.62 (11.84)	82.70 (11.87)	77.75 (11.19)	< 0.001
HbA1c,mean(SD),%	8.64 (2.18)	8.66 (2.20)	8.62 (2.16)	0.802
HDL,mean (SD), mmol/L	1.19 (0.29)	1.10 (0.26)	1.30 (0.30)	< 0.001
LDL, mean (SD), mmol/L	3.06 (0.90)	3.04 (0.86)	3.08 (0.94)	0.469
TC,mean (SD), mmol/L	4.82 (1.18)	4.73 (1.16)	4.94 (1.19)	0.007
TG,mean (SD), mmol/L	2.19 (2.46)	2.40 (2.80)	1.90 (1.85)	0.001
Glu,mean (SD), mmol/L	9.41 (4.03)	9.59 (4.09)	9.16 (3.92)	0.083
GFR,mean(SD), L/min/1.73m^2^	96.80 (20.20)	91.26 (20.58)	104.51 (16.89)	< 0.001
IMT, mean (SD), mm	0.79 (0.24)	0.80 (0.25)	0.78 (0.23)	0.213
Oral antidiabetic agents, n(%)	603 (57.93%)	349 (57.69%)	254 (58.26%)	0.779
lipid-lowering agents, n (%)	252 (25.98%)	137 (24.16%)	115 (28.54%)	0.145
Insulin, n (%)	324 (31.12%)	182 (30.08%)	142 (32.57%)	0.431

*SBP* Systolic blood pressure, *DBP* diastolic blood pressure, *HbA1c* Glycosylated hemoglobin, *HDL* High density lipoprotein, *LDL* Low density lipoprotein, *TC* total cholesterol, *TG* Triglyceride, *Glu* Fasting blood glucose, *BMI* Body mass index, *IMT* carotid intima-media thickness, *GFR* glomerular filtration rate

^a^Data is presented as mean (SD) for continuous variables and as number (%) for categorical variables

### Association between the variation of HbA1c and the changes of IMT

Both linear and non-linear models were used to analyze the association between the variation of HbA1c and the changes of IMT. The mean SD, CV, ARV, VIM of HbA1c was 1.00 ± 0.77, 0.13 ± 0.10, 0.85 ± 0.66, 0.97 ± 0.72, respectively.

Table 2 was the result of multiple linear regressions and the value of β described the association between the variation of HbA1c and ΔIMT%. Except for ARV, the other variation variables (SD, CV, VIM) of HbA1c were associated with ΔIMT% (P < 0.05).

**Table 2:** Association between IMT and variation of HbA1c

	Model1			Model2			Model 3		
	β	95%CI	P	β	95%CI	P	β	95%CI	P
SD	3.46	(1.23, 5.70)	0.002	3.64	(1.27, 6.01)	0.003	3.06	(0.59, 5.53)	0.015
CV	34.35	(16.47, 52.24)	< 0.001	36.04	(17.09, 54.99)	< 0.001	31.99	(12.37, 51.61)	0.001
ARV	1.99	(−1.61, 5.50)	0.266	1.64	(−2.71, 6.00)	0.459	1.15	(−1.61, 3.94)	0.417
VIM	5.15	(2.72, 7.58)	< 0.001	5.37	(2.81, 7.93)	< 0.001	4.87	(2.24, 7.51)	< 0.001

Model 1 adjusted by age, duration of diabetes and sex

model 2 adjusted by age, duration of diabetes, ideal smoking, drink status, sex, BMI, education level, SBP,LDL, HDL, and TC

model 3 adjusted by age, duration of diabetes, ideal smoking, drink status, sex, BMI, education level, SBP,LDL, HDL, TC, Oral antidiabetic agents, lipid-lowering agents and insulin

*CV* coefficient variation, *SD* standard deviation, *ARV* average real variability, *VIM* variability independent of the mean

**Fig. 2:**
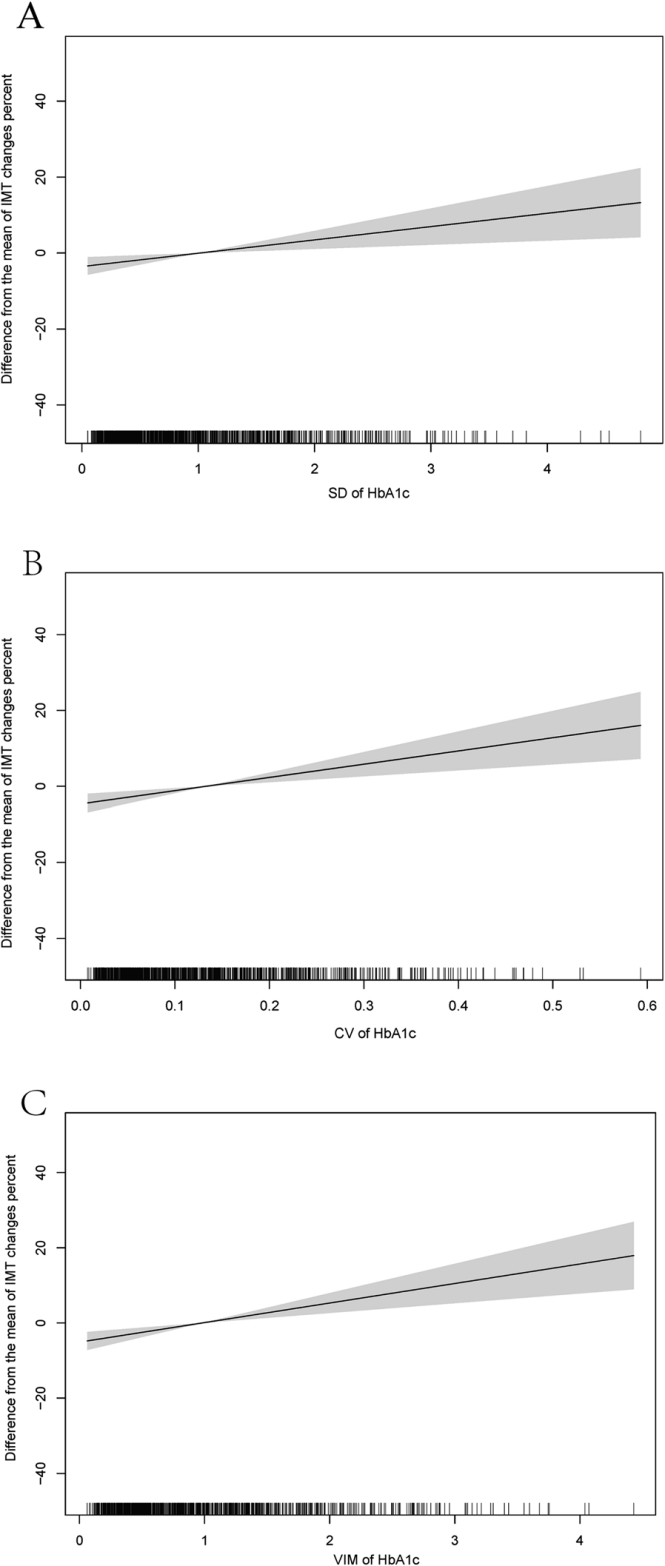
The non-linear association between the variation of HbA1c and the change of IMT.** A** Results of SD;** B** Results of CV;** C** Results of VIM. The generalized addition model was adjusted by age, duration of diabetes, SBP, HDL, LDL, TC, sex, ideal smoking, and drink status. *CV* coefficient variation, *SD* standard deviation, *VIM* variability independent of the mean

The results of GAMs were shown in Fig. 2, the variation of HbA1c (SD, Fig. 2A; CV, Fig. 2B; VIM, Fig. 2C) present a positive relationship with ΔIMT%.

### Trajectory analysis of HbA1c

**Fig. 3:**
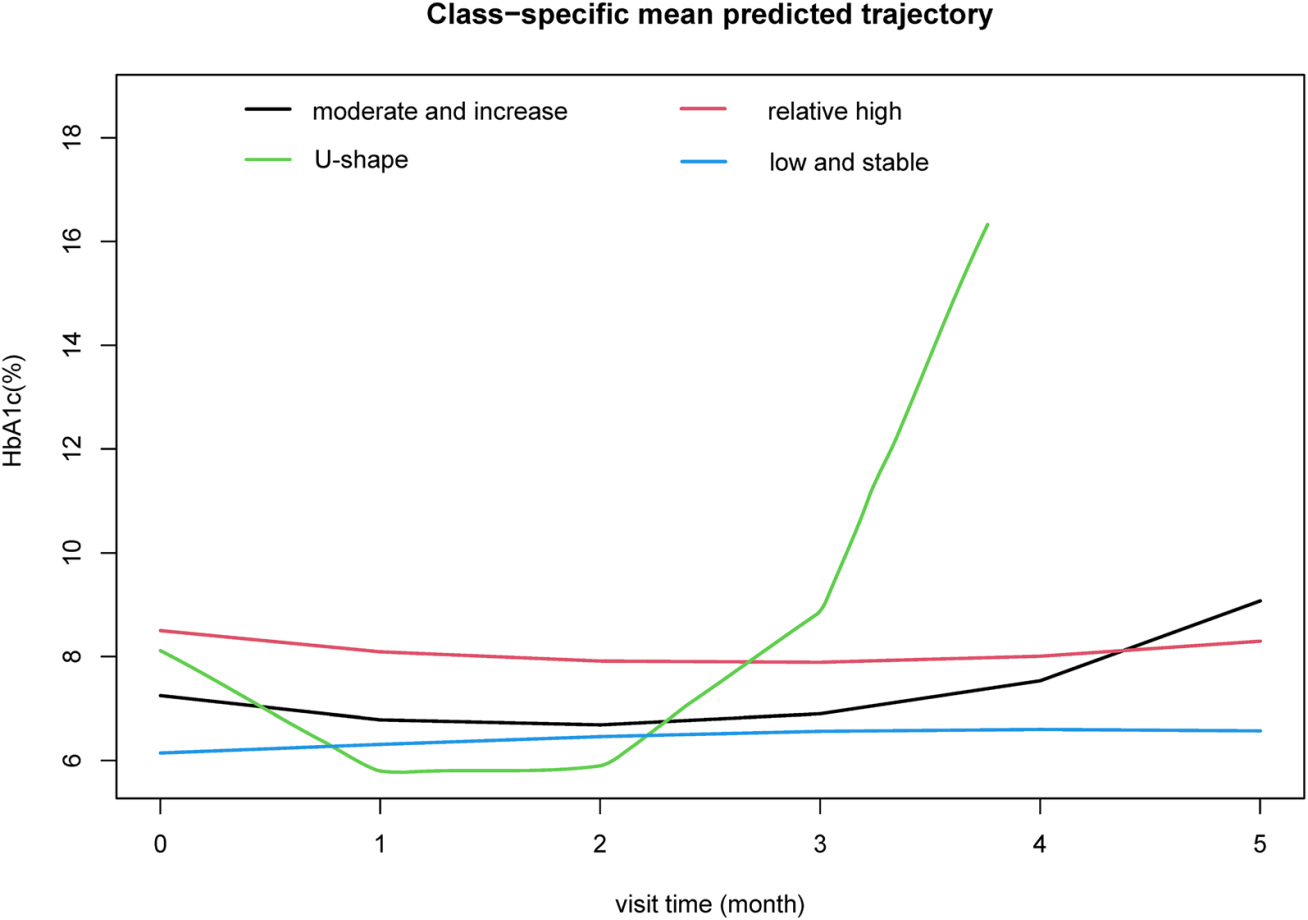
The HbA1c trajectory groups during the follow-up. Low and stable, characterized by maintaining low HbA1c levels throughout follow-up; U-shape, the HbA1c was decreased first and reached the bottom at around two years, then the HbA1c increased during the next following time; Moderate-decrease, starting with an average HbA1c level and experiencing a slight decrease; Relative-high, maintaining high HbA1c levels throughout follow-up

The LCMM identified four discrete trajectory groups of HbA1c (Fig. 3) according to the BIC (The selected model’s BIC was 38559.8). The trajectory groups were named based on baseline HbA1c levels and the visual change patterns of HbA1c over the period of follow-up: (1) low-stable, characterized by maintaining low HbA1c levels throughout the whole period of follow-up; (2) U-shape, the HbA1c was decreased first and reach the bottom, then the HbA1c increased during the next period; (3) moderate and increase, starting with a moderate HbA1c level and experiencing a slight increase over the period of follow-up; (4) Relative high, characterized by maintaining high HbA1c levels throughout follow-up.

Supplemental Table 1 described the statistical characteristics of the four trajectory groups. The low stable trajectory group has the minimum mean value of HbA1c (6.27%) compared with the other 3 trajectory groups. Meanwhile, the U-shape trajectory group has the maximum CV of HbA1c (0.307) compared with the other 3 trajectory groups.

Supplemental Table 2 shows the demographic characteristics of each HbA1c trajectory group at the baseline. Compared to the participants in the first trajectory group of HbA1c, those in the 2nd to 4th trajectory group (Moderate-increase, U-shape, Relative- high, respectively) of HbA1c had a higher level of HbA1c, fasting blood glucose, TG, TC, and LDL (all P < 0.05).

Table 3 describes the covariate-adjusted means (SE) of ΔIMT and ΔIMT%. Comparing with the low stable trajectory group of HbA1c, the covariate-adjusted means (SE) of ΔIMT% were significantly higher in Moderate-increase, U-shape and relative high trajectory groups, the mean (SE) were 7.03 (0.031), 15.49 (0.185), 14.15 (0.029), respectively.

**Table 3:** Covariate-adjusted means of change of IMT by HbA1c trajectory group

Trajectory group	Participants n (%)	Change of IMT, mean (SE), cm/s	Change of IMT, mean (SE), %	P value	P for trend
HbA1c	1041				0.001
low stable	148	−0.001 (0.001)	−0.166 (0.116)	NA	
Moderate and increase	253	0.054 (0.000)	7.03 (0.031)	< 0.001	
U shape	81	0.106 (0.001)	15.49 (0.185)	< 0.001	
relative higher	559	0.114 (0.001)	14.15 (0.029)	< 0.001	

*IMT* carotid intima-media thickness

Mode l adjusted by age, duration of diabetes, ideal smoking, drink stauts, sex, BMI, education level, SBP,LDL, HDL, TC, Oral antidiabetic agents, lipid-lowering agents and insulin

### Cross-lagged panel model of HbA1c and IMT

**Fig. 4:**
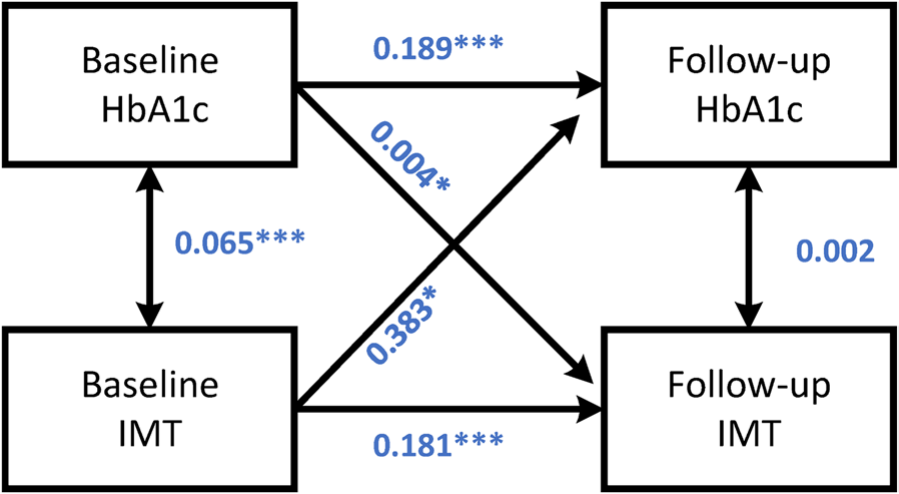
The cross-lagged panel analyses of HbA1c and IMT during at baseline and follow-up. Model adjusted by age, sex, duration of diabetes, SBP, smoke, drink, HDL, LDL, Oral antidiabetic agents, lipid-lowering agents and insulin.*p < 0.05;**p < 0.01;***.p < 0.001. *IMT* carotid intima-media thickness, *HbA1c* Glycosylated hemoglobin

The results of cross-lagged panel models were shown in Fig. 4, there were significant bidirectional cross-lagged associations between HbA1c and IMT after adjusting for covariates. The standardized correlation coefficient of baseline HbA1c and follow-up IMT (β1) was 0.004 (95%CI 0.001–0.008, P = 0.046), and that of baseline IMT and follow-up HbA1c (β2) was 0.383 (95%:0.035–0.732, P = 0.031). The standardized mean square residual (SRMR) and comparative fit index (CFI) were 0.057 and 0.606, respectively. More details of the CLPM could be found in Supplemental Table 3.

### Subgroup analysis

We also performed sex subgroup analyses to investigate the consistency of the association between variation of HbA1c and the changes of IMT.

The result of Multiple linear regressions in the sex group was shown in Supplemental Table 4. Except for ARV, the other variation variables (SD, CV, VIM) of HbA1c were associated with ΔIMT% both in males and females, which was consistent with the result of all participants.

## Discussion

In this study, we described the HbA1c pattern in Chinese diabetes patients in a long-term follow-up period and determined the causal relationship between glycemic control patterns and atherosclerosis progression, providing high-quality evidence for controlling diabetes complications.

We used some variability indicators to illustrate the HbA1c pattern. These indicators are commonly used in cardiovascular disease research to describe the variability of systolic blood pressure [21, 22]. In our study, most of the variability indicators (SD, CV, VIM) of HbA1c were positively associated with ΔIMT% (P < 0.05). The indicator of ARV considered the temporality of HbA1c measurement in the calculation procedure, while it did not reflect the changing trend of HbA1c, which was the reason the analysis of ARV did not get the expected results. Our results were similar to Fang’s study, which explored the relationship between HbA1c variability and IMT. In addition, the results of subgroup analysis in the sex group were consistent with the main analysis, which indicated the positive relationship between HbA1c variability and changes in IMT was not influenced by sex.

Only the absolute value of a single time point, and variability of values may not reflect the patterns of HbA1c controlling comprehensively when interpreting blood glucose data. So, we used the trajectory analysis method to describe the different clustering patterns of HbA1c control in diabetes patients [14]. Compared with traditional longitudinal data analysis methods, this method can better understand the etiology association by analyzing the phenotype of certain "high-risk" subgroups [23, 24]. In the trajectory analysis procedure, four trajectory groups of HbA1c were identified, which was consistent with the clinical experience of diabetes patients. Moreover, we drew the statistical characteristics of the four trajectory groups. The U-shape trajectory group of diabetes patients showed a great variation of HbA1c (the CV and SD were 0.307 and 2.11, respectively), meanwhile, the covariate-adjusted means (SE) of ΔIMT% were also the largest in the U-shape trajectory group, which was consistent with results of GAM in this study. These results indicated that except for high levels of blood glucose, the variability of glycemic level is also a risk factor for the progression of diabetic atherosclerosis. Our study suggests that patients with diabetes should avoid roller-coaster changes of blood glucose during a long period of glycemic level control.

The cross-lag panel model (CLPM) is a statistical method used to solve the problem of the causal effect of cross-lag correlation data in longitudinal studies. We used the CLPM to explore the temporal and bidirectional associations between long-term measurements of HbA1c and IMT based on a Chinese population. In the cross-lagged analyses, the baseline HbA1c was shown to have a temporal relationship with subsequent atherosclerosis, which verified the causal relationship of HbA1c and atherosclerosis. Meanwhile, the baseline IMT and follow-up HbA1c also have a positive relationship temporally. Similar results for the relation between glycemic level and arterial stiffness was reported in the previous study [25]. The CLPM results complement the study theme and describe the relationship between baseline HbA1c and changes in IMT (progression of arteriosclerosis) at long-term follow-up. In clinical practice, we found that patients with higher blood glucose levels in their first visit may have difficulty controlling their blood glucose and were at higher risk for blood glucose fluctuations, which was consistent with the results of this study (Supplemental Table 1–2). The fluctuation of blood glucose needs to be quantified, so we use the variability indexes and trajectory analysis to comprehensively describe the differences in blood glucose control. Moreover, due to the missing value of IMT in the middle period of follow-up, we could not get enough results of IMT in visit -to-visit. The random intercept cross-lagged panel model (RI-CLPM) could not be established, which could better illustrate the casual relationship between HbA1c and IMT.

Several potential mechanisms may expound the association between the HbA1c pattern and the progression of atherosclerosis. Firstly, endothelial dysfunction may lead to impaired relaxation, contraction, or sparse distribution of capillaries, leading to arterial wall sclerosis. Arteriosclerosis can cause damage to capillaries, forming a vicious cycle [26]. Elevated FPG levels could induce oxidative stress, alter protein kinase signaling, and trigger certain miRNA and epigenetic modifications, which were regarded as another possible pathogenesis in the atherosclerosis process [27]. In addition, Atherosclerosis and arteriosclerosis have similar risk factors. Microvascular damage from significant artery stiffness (LAS) may affect glucose homeostasis. A recent mendelian randomization study demonstrated a two-way causal relationship between glucose homeostasis and arterial stiffness [28].

Our study determined the causal relationship between glycemic control patterns and atherosclerosis progression; however, we could not give more suggestion about the effects of the new treatments for diabetes. The recent study revealed a decreased risk of coronary heart disease in patients with type 2 diabetes treated with GLP-1 receptor agonists and SGLT2 inhibitors, which provided the idea for our next research [29, 30].

The main strength of this study was that MMC was a national project, standard management of metabolic patients, and rigorous and comprehensive risk factor measurements were collected, meaning that the model could be adjusted for more potentially confounding variables. Secondly, we had full consideration for individual and inter-individual differences in blood glucose control when interpreting the blood glucose data. Thirdly, in long-term surveys of glycemic level tracking, fasting blood glucose is not a reliable indicator, as it can change in a short period due to diet, exercise, and environmental factors [2, 31]. The glycated hemoglobin used in this study can reflect the average blood glucose level over a period, making the research results more accurate [32].

This study also has some limitations. Firstly, the sample size was relatively insufficient, so we could not explore more subgroup analyses such as education level, and age group. Secondly, the participants in the U-shape group are younger and have a shorter duration of diabetes, which is consistent with the features of latent autoimmune diabetes in adults (LADA). However, we could not exclude these participants due to the limit of inspection results. Thirdly, patients may decrease the visit times of MMC during the prevalence of COVID-19, which may cause some participants to lose follow-up. Lastly, the mean follow-up time of this study was 28.78 months; the conclusion may be more powerful with a longer period of follow-up. Finally, our results in this single cohort study need further validation in other populations.

## Conclusion

We found four discrete trajectory groups of HbA1c during the long-term follow-up of diabetes. There was a positive association between HbA1c variability and the progression of atherosclerosis. Our study suggested that patients with diabetes should avoid roller coaster changes in blood glucose over a long period when controlling blood glucose.

### Supplementary Information

Supplementary material 1.

## Data Availability

Obtained with the approval of corresponding author.

## Abbreviations

SBP: Systolic blood pressure
HbA1c: Glycosylated hemoglobin
HDL: High-density lipoprotein
LDL: Low-density lipoprotein
TC: Total cholesterol
TG: Triglyceride
Glu: Fasting blood glucose
BMI: Body mass index
DN: Diabetic nephropathy DN
CVD: Cardiovascular disease
GAMs: The generalized additive models
ΔIMT: The changes of carotid intima-media thickness
CLPM: Cross-lagged panel model
LCMM: The latent class mixture model
