# Supplementary material for: The relationship between HbA1c control pattern and atherosclerosis progression of diabetes: a prospective study of Chinese population
Source: Diabetol Metab Syndr. 2024 Jun 10;16:127. doi: 10.1186/s13098-024-01370-4 (PMC11163799; doi:10.1186/s13098-024-01370-4)
Supplement: Supplementary file 1 — Supplementary material 1. [file 13098_2024_1370_MOESM1_ESM.docx]

**Table S1. Statistics characters of HbA1c in each trajectory groups**

| **class** | **Mean** | **SD** | **CV** | **Max** | **min** |
| --- | --- | --- | --- | --- | --- |
| **Low stable** | 6.27 | 0.73 | 0.116 | 13.6 | 2.3 |
| **Moderate and increase** | 6.72 | 1.07 | 0.159 | 15.9 | 4.9 |
| **U shape** | 6.87 | 2.11 | 0.307 | 15.6 | 3.9 |
| **Relative higher** | 8.07 | 1.53 | 0.189 | 18.7 | 5.0 |

Abbreviation: CV, coefficient variation; SD, standard deviation.

**Table S2. Demographic characteristics of participants in each trajectory group at baseline line**

|  | **ALL** | **Low stable** | **Moderate and increase** | **U shape** | **Relative high** | **p** | **p.trend** |
| --- | --- | --- | --- | --- | --- | --- | --- |
|  | ***N=1041*** | ***N=148*** | ***N=253*** | ***N=81*** | ***N=559*** |  |  |
| **Duration,mean(SD),month** | 84.06 (84.96) | 71.67 (78.01) | 62.05 (73.80) | 19.04 (44.17) | 107.07 (87.73) | <0.001 | <0.001 |
| **Age,mean(SD),year** | 51.27 (11.81) | 50.41 (10.95) | 50.64 (11.39) | 41.77 (11.77) | 53.15 (11.52) | <0.001 | 0.001 |
| **Ideal Smoking,n(%)** | 315 (30.41%) | 40 (27.03%) | 81 (32.40%) | 35 (43.21%) | 159 (28.55%) | 0.037 | 0.756 |
| **Drink status,n(%)** | 435 (42.07%) | 60 (40.82%) | 113 (45.20%) | 35 (43.21%) | 227 (40.83%) | 0.683 | 0.549 |
| **High school or more, n (%)** | 625 (60.04%) | 101 (68.24%) | 153 (60.47%) | 62 (76.54%) | 309 (55.28%) | <0.001 | 0.005 |
| **Weight,mean(SD),kg** | 74.52 (14.19) | 72.11 (12.06) | 75.17 (12.98) | 82.01 (19.43) | 73.78 (14.02) | <0.001 | 0.794 |
| **BMI,mean(SD),kg/m2** | 26.82 (4.00) | 26.02 (3.68) | 26.97 (3.93) | 27.89 (5.04) | 26.82 (3.91) | 0.007 | 0.189 |
| **height,mean(SD),cm** | 166.34 (8.62) | 166.34 (8.88) | 166.77 (7.92) | 170.87 (8.59) | 165.49 (8.66) | <0.001 | 0.071 |
| **SBP,mean(SD),mmHg** | 133.34 (18.00) | 133.79 (18.24) | 133.80 (17.85) | 130.12 (16.54) | 133.47 (18.21) | 0.412 | 0.795 |
| **DBP,mean(SD),mmHg** | 80.62 (11.84) | 81.65 (12.08) | 81.77 (11.97) | 82.57 (9.50) | 79.55 (11.94) | 0.017 | 0.007 |
| **Sex,male,n(%)** | 605 (58.12%) | 93 (62.84%) | 148 (58.50%) | 61 (75.31%) | 303 (54.20%) | 0.002 | 0.071 |
| **HbA1c,mean(SD),%** | 8.64 (2.18) | 6.12 (0.62) | 8.01 (1.92) | 10.68 (1.82) | 9.30 (1.93) | <0.001 | <0.001 |
| **HDL,mean (SD), mmol/L** | 1.19 (0.29) | 1.24 (0.28) | 1.21 (0.30) | 1.05 (0.29) | 1.18 (0.29) | <0.001 | 0.018 |
| **LDL, mean (SD), mmol/L** | 3.06 (0.90) | 2.74 (0.78) | 3.09 (0.89) | 3.27 (0.96) | 3.10 (0.90) | <0.001 | 0.002 |
| **TC,mean (SD), mmol/L** | 4.82 (1.18) | 4.44 (0.97) | 4.85 (1.16) | 5.02 (1.28) | 4.87 (1.20) | <0.001 | 0.003 |
| **TG,mean (SD), mmol/L** | 2.19 (2.46) | 1.64 (1.39) | 2.08 (2.40) | 2.81 (2.59) | 2.30 (2.65) | 0.003 | 0.008 |
| **Glu,mean (SD), mmol/L** | 9.41 (4.03) | 6.93 (1.71) | 8.77 (3.33) | 10.11 (4.58) | 10.25 (4.34) | <0.001 | <0.001 |
| **GFR,mean(SD),mL/min/1.73m2** | 96.80 (20.20) | 90.42 (20.67) | 96.51 (20.42) | 107.07 (16.22) | 97.14 (19.89) | <0.001 | 0.004 |
| **IMT, mean (SD), mm** | 0.79 (0.24) | 0.81 (0.41) | 0.78 (0.19) | 0.69 (0.19) | 0.81 (0.21) | <0.001 | 0.477 |
| **Oral antidiabetic agents, n (%)** | 603 (57.93%) | 111 (75.00%) | 180 (71.15%) | 33 (40.74%) | 279 (49.91%) | <0.001 | <0.001 |
| **lipid-lowering agents, n (%)** | 252 (25.98%) | 34 (25.76%) | 59 (25.21%) | 10 (12.35%) | 149 (28.49%) | 0.022 | 0.324 |
| **Insulin, n (%)** | 324 (31.12%) | 23 (15.54%) | 41 (16.21%) | 25 (30.86%) | 235 (42.04%) | <0.001 | <0.001 |

Abbreviation: SBP, Systolic blood pressure; DBP, diastolic blood pressure; HbA1c, Glycosylated hemoglobin; HDL, High density lipoprotein; LDL, Low density lipoprotein; TC, total cholesterol; TG, Triglyceride; Glu, Fasting blood glucose; BMI, Body mass index; IMT, carotid intima-media thickness; GFR, glomerular filtration rate.

**Table S3. Adjusted cross-lagged standard regression coefficient of IMT and HbA1c**

|  | **HbA1c1→IMT2** | **IMT1→HbA1c2** | **HbA1c1→HbA1c2** | **IMT1→IMT2** | **R2 of HbA1c** | **R2 of IMT** | **CFI** | **SRMR** | **RMSER** |
| --- | --- | --- | --- | --- | --- | --- | --- | --- | --- |
| **Model 1** | 0.005(0.001,0.009)** | 0.376(0.020,0.731)* | 0.213(0.173,0.252)*** | 0.183(0.149,0.217)*** | 0.109 | 0.226 | 0.737 | 0.094 | 0.196 |
| **Model 2** | 0.006(0.003.0.010)** | 0.369(0.020,0.718)* | 0.221(0.181,0.260)*** | 0.185(0.151,0.220)*** | 0.176 | 0.242 | 0.752 | 0.070 | 0.118 |
| **Model 3** | 0.005(0.001,0.009)* | 0.378(0.027,0.728)* | 0.216(0.176,0.255)*** | 0.181(0.146,0.216)*** | 0.174 | 0.243 | 0.681 | 0.068 | 0.122 |
| **Model 4** | 0.004(0.001,0.008)** | 0.383(0.035,0.732)* | 0.189(0.150,0.228)*** | 0.181(0.146,0.215)*** | 0.167 | 0.244 | 0.606 | 0.057 | 0.119 |

Model 1 adjusts by age and sex.

Model 2 adjusts by age, sex, duration of diabetes, SBP, ideal smoking and drink status.

Model 3 adjusts by age, sex, duration of diabetes, SBP, ideal smoking, drink status, HDL, and LDL.

Model 4 adjusts by age, sex, duration of diabetes, SBP, ideal smoking, drink status, HDL, LDL, Oral antidiabetic agents, lipid-lowering agents and insulin.

**Table S4. Association between IMT and variation of HbA1c in sex groups.**

|  | **male** | | | **female** | | |
| --- | --- | --- | --- | --- | --- | --- |
|  | **β** | **95%CI** | **P** | **β** | **95%CI** | **P** |
| **SD** | 3.40 | (0.40,6.40) | 0.027 | 3.98 | (0.027,7.94) | 0.049 |
| **CV** | 34.19 | (10.43,57.95) | 0.005 | 39.57 | (7.39,71.74) | 0.016 |
| **ARV** | 1.63 | (-0.84,4.21) | 0.215 | 1.84 | (-0.87,4.55) | 0.187 |
| **VIM** | 5.09 | (1.89,8.28) | 0.002 | 6.00 | (1.59,10.41) | 0.008 |

Model adjusted by age, ideal smoking, drink status, sex, BMI, education level, SBP, LDL, HDL, and TC.

Abbreviation: CV, coefficient variation; SD, standard deviation; ARV, average real variability; VIM, variability independent of the mean.
